# Supplementary material for: Ferrostatin-1 inhibits fibroblast fibrosis in keloid by inhibiting ferroptosis
Source: PeerJ. 2024 Jun 14;12:e17551. doi: 10.7717/peerj.17551 (PMC11182022; doi:10.7717/peerj.17551)
Supplement: Supplemental Information 1 [file peerj-12-17551-s001.docx]

Figure1C

SLC7A11 (xCT)









GPX4









TFRC









Nrf2









GAPDH









Figure2C

SLC7A11 GPX4 TFRC









Nrf2 GAPDH







Figure3B

SLC7A11 GPX4 GAPDH









Figure3C

TFRC Nrf2 GAPDH









Figure4A

a-SMA collagen I collagen III









GAPDH





Figure5B

SLC7A11 GPX4 GAPDH









Figure5C

TFRC Nrf2 GAPDH









Figure6A

a-SMA collagen I collagen III









GAPDH
